# Quantification of two isomeric flavones in rat colon tissue using reverse phase high performance liquid chromatography

**DOI:** 10.1186/s13104-016-2358-y

**Published:** 2017-01-07

**Authors:** Crystal L. Whitted, Victoria E. Palau, Ruben D. Torrenegra, Oscar E. Rodriguez, Sam Harirforoosh

**Affiliations:** 1 Department of Pharmaceutical Sciences, Gatton College of Pharmacy, East Tennessee State University, Box 70594, Johnson City, TN 37614-1708 USA; 2 Universidad de Ciencias Aplicadas y Ambientales, Bogotá, Colombia; 3 Department of Environmental Engineering, Faculty of Engineering, Universidad El Bosque, Bogotá, Colombia

**Keywords:** 5,7-Dihydroxy-3,6,8-trimethoxy flavone, 3,5-Dihydroxy-6,7,8-trimethoxy flavone, HPLC, Colon, Flavonoids, Cancer

## Abstract

**Background:**

Antineoplastic activity has been previously shown for two isomeric flavones, 5,7-dihydroxy-3,6,8-trimethoxy flavone (flavone A) and 3,5-dihydroxy-6,7,8-trimethoxy flavone (flavone B), against colon cancer cell lines (Thomas et al. in PLoS ONE 7:e39806, 5). Here, we present modified methods for the extraction and quantification of flavones A and B in rat colon tissue after intravenous dosing via high performance liquid chromatography, from the originally described procedure for extraction and quantification in rat plasma (Whitted et al. in J Chromatogr B Analyt Technol Biomed Life Sci 1001:150–155, 7).

**Results:**

Modifications included tissue homogenization (1 g tissue: 2 mL water), filtration of the supernatant with a PVDF membrane, and the use of only one calibration curve to determine the concentration of each flavone in colon tissue. Good separation was achieved and representative equations were linear with *r* ^*2*^ ≥ 0.99 for both flavones. Precision and accuracy for flavone A ranged from 0.88–24.03 and 109–116%. Precision and accuracy for flavone B ranged from 1.62–33.56 and 98–113%. Concentrations of 1639 ± 601 ng/g flavone A and 5975 ± 2480 ng/g of flavone B were detected in rat colon tissue 6 h post dosing.

**Conclusions:**

Modifications to the extraction methods for flavone A and flavone B from rat colon tissue had good separation, precision, and accuracy.

## Background

Many flavonoids have been recognized for their antitumor properties [1–4]. It is known that these activities are greatly determined by their chemical structure which subsequently dictates their ability to interact with cellular molecules [1]. This differential effect has been observed with flavone isomers 5,7-dihydroxy-3,6,8-trimethoxy flavone (flavone A) and 3,5-dihydroxy-6,7,8-trimethoxy flavone (flavone B) [5]. Specifically, each isomer has been shown to target colon cancer cells with distinct phenotypical characteristics via different mechanisms of action [6]. However, it is unknown whether either of these flavones distribute to the colon after intravenous administration of these compounds. Modifications were made to methods developed for extracting and quantifying these flavones in rat plasma [7] using reverse phase high performance liquid chromatography (HPLC). The modifications included preparation of the sample by homogenization in water, filtration using a polyvinylidene fluoride (PVDF) membrane, and the creation of a single calibration curve to determine the concentration of either flavone in colon tissue.

These methods allowed for the detection of flavone A and flavone B in rat colon. Our data indicate that flavone B was found in higher concentrations in the colon than flavone A, which may be the result of higher volume of distribution value of flavone B [7] compared to that of flavone A.

## Methods

### Procedure to extract and purify flavones A and B

The compounds were obtained as described before [5]. Briefly, flavone A was purified from dried flowers of *Gnaphalium elegans* extracted with chloroform using a silica gel chromatography column. Flavone B was purified from leaves of *Achyrocline bogotensis*, using chloroform, followed by crystallizations in hexane. The physical and spectroscopic properties of these compounds allowed their proper identification.

### Stock solution and standards

Stock solutions of flavone A at a concentration of 100 µg/mL, prepared as described previously [7], and 25 µg/mL celecoxib (Toronto Research Chemicals; Toronto, ON, CA) were prepared with acetonitrile/water/acetic acid/triethylamine (60:40:0.2:0.05). Stock solutions of 100 µg/mL of flavone B, prepared as described previously [7], and 25 µg/mL diclofenac (MP Biomedicals, LLC; Solon, OH) were prepared with acetonitrile/water/acetic acid/trimethylamine (70:30:0.2:0.05). All stock solutions were stored protected from light at 4 °C. HPLC grade acetonitrile, acetic acid, trimethylamine, and water were purchased from Fisher Scientific (Pittsburgh, PA). Flavone A or flavone B were mixed with polyethylene glycol 400 (Electron Microscopy Sciences; Hatfield, PA) for intravenous injection.

### Sample preparation

Colon tissue was homogenized using a PowerGen 700 from Fisher Scientific (Pittsburgh, PA) in a 1:2 ratio with water (1 mg/2 mL). Serial concentrations for calibration curves (flavone A: 250–100,000 ng/g and flavone B: 1000–25,000 ng/g) were prepared. Briefly, 100 μL of blank homogenate was spiked with 100 μL flavone, 100 µL internal standard (25 µg/mL celecoxib or diclofenac), and 200 μL of organic solvent (acetonitrile). The samples were vortex mixed before being centrifuged for 15 min at 3000×*g*. The supernatant was removed and filtered with a PDVF filter (0.45 µm) into a clean tube and evaporated using a Labconco vacuum concentrator (Kansas City, MO). Mobile phase (200 μL) was used to reconstitute the residue and 100 μL of sample was injected into the HPLC column. Analysis was conducted in triplicate.

### HPLC conditions and quantitation

HPLC assays were performed using a Shimadzu liquid chromatography system (Shimadzu Scientific Instruments Inc., Columbia, Maryland, USA) with an ACE C18 (100 × 4.6 mm) (Aberdeen, Scotland) column. Mobile phases used for HPLC contained acetonitrile/water 60:40 (flavone A) and 70:30 (flavone B) with 0.2% acetic acid and 0.05% triethylamine. Detection wavelength was at 245 nm with a temperature of 30 °C. Flow rate was 0.4 mL/min with run times of 11 and 10 min, respectively. LC solutions program was used to collect and analyze the data.

### Animals and drug administration

The methods described here were used to determine the concentrations of flavone A or flavone B in colon tissue collected from male Sprague–Dawley rats (Charles River Laboratories, Raleigh, NC, USA) used in a previous study [7]. Briefly, flavones were mixed in polyethylene glycol 400 and were administered by intravenous injection to deliver a 20 mg/kg dose of flavone A (n = 6) or flavone B (n = 6). Animals were euthanized under anesthesia 6 h post dosing. Colon tissue was collected and flash frozen using dry ice and stored at −80 °C until analyzed for the measurement of concentrations of flavones.

## Results

**Fig. 1:**
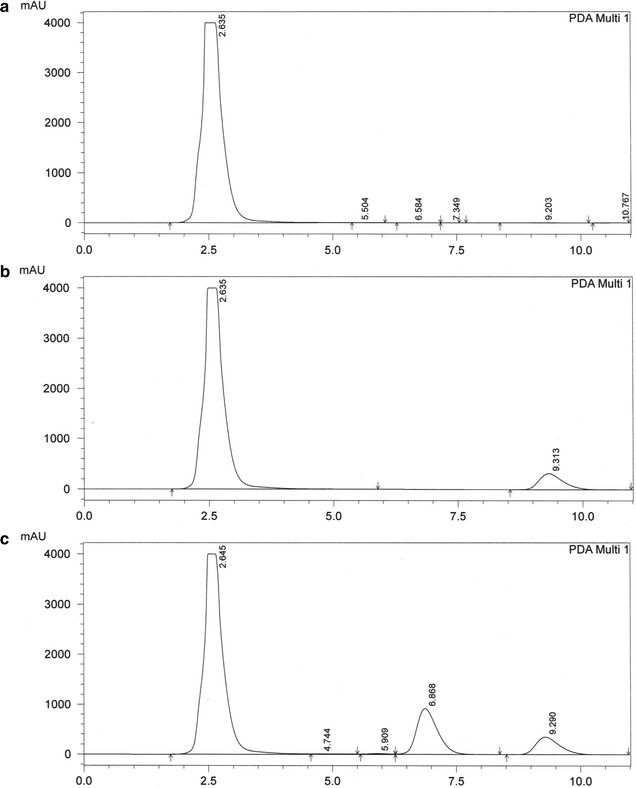
Elution of flavone A from colon. HPLC chromatographs of **a** blank colon; **b** colon spiked with internal standard (celecoxib 25 µg/mL); **c** colon spiked with flavone A (100 µg/mL) and internal standard

**Fig. 2:**
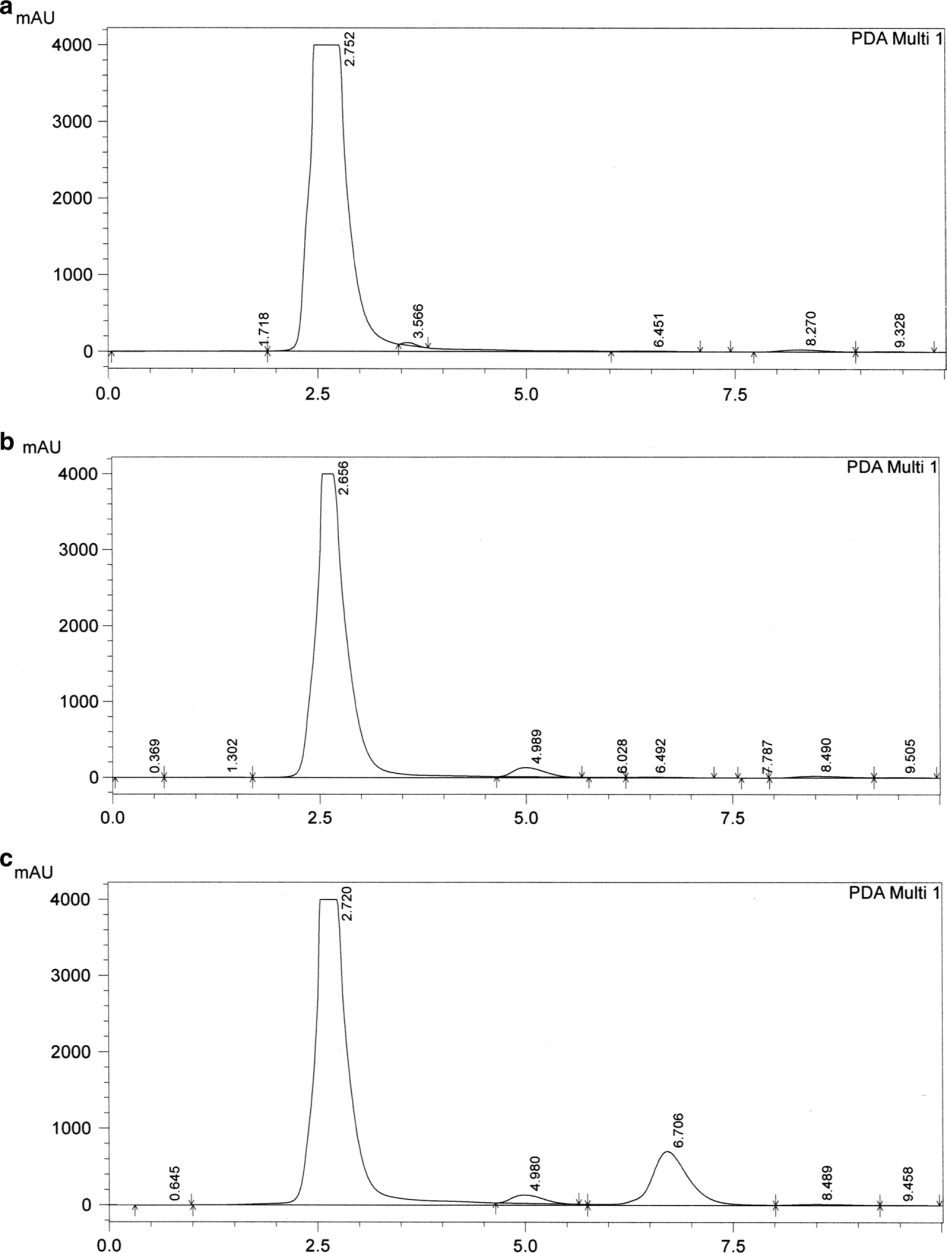
Elution of flavone B from colon. HPLC chromatographs of **a** blank colon; **b** colon spiked with internal standard (diclofenac 25 µg/mL); **c** colon spiked with flavone B (100 µg/mL) and internal standard

**Fig. 3:**
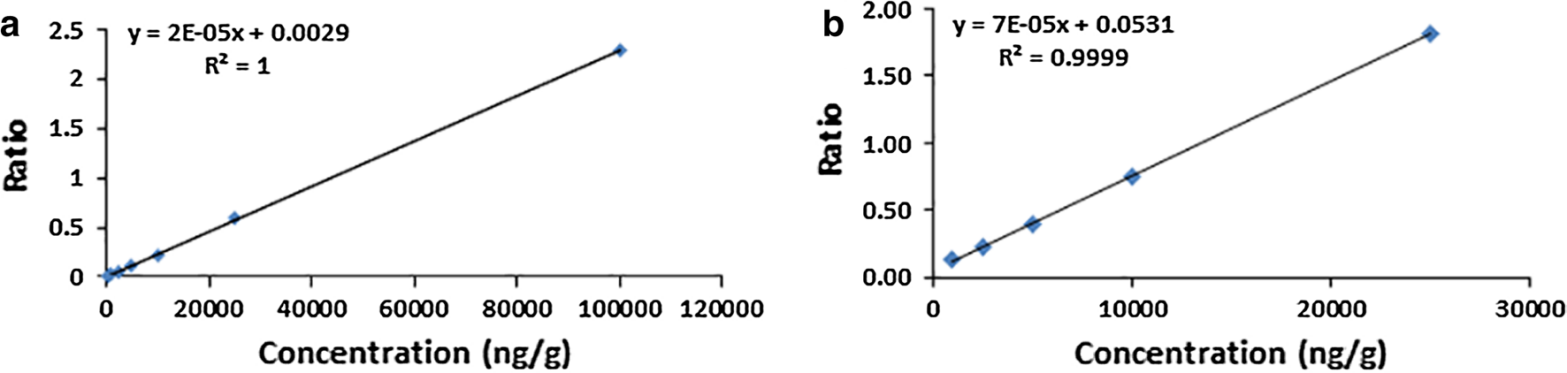
Calibration curves were made by graphing the ratio of flavone/internal standard area under the curve extracted from colon tissue versus the known concentration of flavone. Representative calibration curves for **a** flavone A and **b** flavone B are presented

**Fig. 4:**
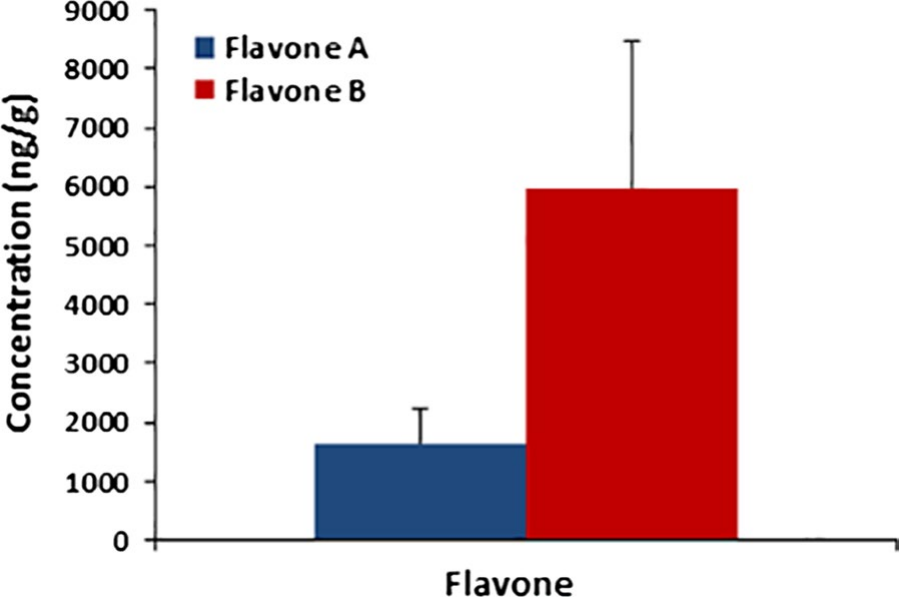
Distribution of flavones to target tissue. Male Sprague–Dawley rats received 20 mg/kg of either flavone A or flavone B via intravenous injection. The colon tissues were collected 6 h after dosing and analyzed for the presence of flavone using a HPLC system

Good separation was achieved (Figs. 1, 2) and the peak area ratios of flavone against internal standard were plotted in Excel to make the calibration curves (Fig. 3). Representative equation for flavone A concentrations 250–100,000 ng/g was y = 2E − 05x + 0.0029 and flavone B concentrations 1000–25,000 ng/g was y = 7E − 05x + 0.0531 with *r* ^*2*^ ≥ 0.99. Three calibration curves were used to determine the precision (coefficient of variation—CV) and accuracy of the methods. Data is presented as mean ± standard deviation (Tables 1, 2). Analysis yielded 1639 ± 601 ng/g of flavone A and 5975 ± 2480 ng/g of flavone B in colon tissue (Fig. 4).

**Table 1:** Precision and accuracy for 250–100,000 ng/g flavone A extracted from rat colon tissue

Concentration (ng/g)	Error (%)	Observed (n = 3)	CV	Accuracy (%)
100,000	13.88 ± 2.14	113,888 ± 2138	1.87	114
25,000	16.32 ± 1.06	29,081 ± 265	0.91	116
10,000	11.83 ± 4.15	11,182 ± 415	3.71	111
5000	9.93 ± 0.97	5496 ± 48	0.88	109
2500	13.92 ± 4.87	2848 ± 121	4.27	113
1000	11.45 ± 8.90	1114 ± 89	7.98	111
500	16.65 ± 21.51	558 ± 129	23.21	111
250	21.02 ± 19.30	285 ± 68	24.03	113

**Table 2:** Precision and accuracy for 1000–25,000 ng/g flavone B extracted from rat colon tissue

Concentration (ng/g)	Error (%)	Observed (n = 3)	CV	Accuracy (%)
25,000	1.51 ± 0.50	25,204 ± 410	1.62	101
10,000	2.89 ± 0.52	10,075 ± 345	3.43	101
5000	3.69 ± 3.42	4934 ± 271	5.51	98
2500	4.22 ± 2.55	2480 ± 142	5.73	99
1000	25.18 ± 27.00	1128 ± 378	33.56	113

## Conclusion

Modifications to the methods developed for extraction and quantification of flavone A and flavone B from rat colon tissue yield good separation, precision, and accuracy. The distribution of both flavones to the colon suggests that they would be good candidates for in vivo antitumor studies for colon cancer.

## Abbreviations

Flavone A: 5,7-dihydroxy-3,6,8-trimethoxy flavone
Flavone B: 3,5-dihydroxy-6,7,8-trimethoxy flavone
HPLC: high performance liquid chromatography
PVDF: polyvinylidene fluoride
